# Substrate Designs for Stable Potassium Metal Anodes

**DOI:** 10.1021/acsaem.5c02661

**Published:** 2025-10-22

**Authors:** Yupei Han, Yang Xu

**Affiliations:** Department of Chemistry, 4919 University College London, 20 Gordon Street, London WC1H 0AJ, U.K.

**Keywords:** potassium metal batteries, substrate engineering, dendrite suppression, nucleation and deposition, scalable anode design

## Abstract

Potassium metal batteries (PMBs) are gaining attention as low-cost, sustainable, and high-energy storage. Their practical implementation, however, is impeded by instability of the potassium (K) metal anode, manifested as dendritic growth, large volume fluctuations, and fragile solid electrolyte interphases (SEIs), all of which accelerate capacity fading and safety risks. This review highlights recent advances in substrate design for stabilizing K metal anodes, categorized into five strategies: (i) three-dimensional host architectures, (ii) heteroatom doping and molecular grafting, (iii) inorganic nanoparticle incorporation, (iv) alloying seed engineering, and (v) substrate-regulated SEI formation via work function modulation. Mechanistic insights from experimental and theoretical studies are integrated with performance comparisons to evaluate trade-offs between deposition control, SEI stability, scalability, and cost. Key challenges for commercialization are outlined, including long-term cycling under practical conditions, integration with high-energy-density cathodes, and scalable fabrication. By advancing structural, chemical, and electronic design principles, PMBs can progress toward reliable, high-performance energy storage.

## Graphical abstract

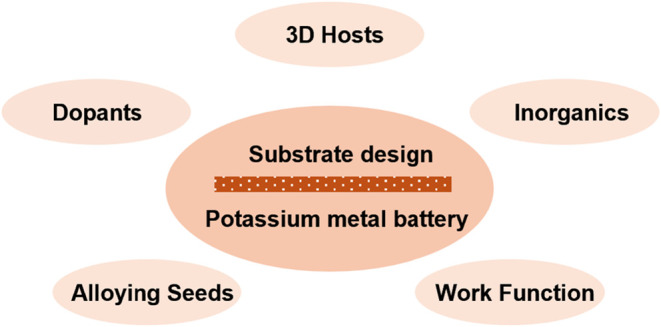

## 1 Introduction

Achieving global carbon neutrality requires not only the rapid expansion of renewable energy generation but also the deployment of efficient, reliable, and sustainable energy storage systems to balance supply demand fluctuations.^1,2^ Rechargeable batteries have become a cornerstone of zero-emission technologies. Among them, lithium-ion batteries (LIBs) have achieved widespread adoption in electric transportation, portable electronics, unmanned aerial vehicles, and robotics, owing to their high energy density, low self-discharge rate, and long cycle life.^3^ However, both the cathode and anode capacities in LIBs are approaching their theoretical limits, hindering progress toward achieving the target energy density of 500 Wh kg^–1^.^4^

Lithium metal batteries (LMBs), which employ energy-dense lithium (Li) metal anodes, offer a promising route to reach this benchmark at the device level, including packaging.^5,6^ Nevertheless, the scarcity of Li in the upper crust (0.002 wt %),^7^ uneven geographical distribution,^8^ and the surging demand from battery production (∼87% of global Li consumption),^9^ raise concerns over long-term resource availability.^10^ Pronounced price volatility and high resource costs (∼10^4^ $ per metric ton)^9^ further restrict their suitability for large-scale, cost-sensitive applications such as grid-level storage.

To meet net-zero targets, next-generation rechargeable battery chemistries must combine high performance with the use of earth-abundant, low-cost materials.^11,12^ Inexpensive sodium- and potassium-ion batteries are attracting increasing attention.^13–15^ To further enhance energy density, potassium metal batteries (PMBs) have recently emerged as a promising contender, owing to potassium (K)’s upper-crustal abundance (2.32 wt %, over 1000 times that of Li),^7^ wide geographic availability, low resource cost (∼10^3^ $ per metric ton, one-tenth that of Li, Figure 1A),^9^ and favorable electrochemical properties.^16,17^

**Figure 1:**
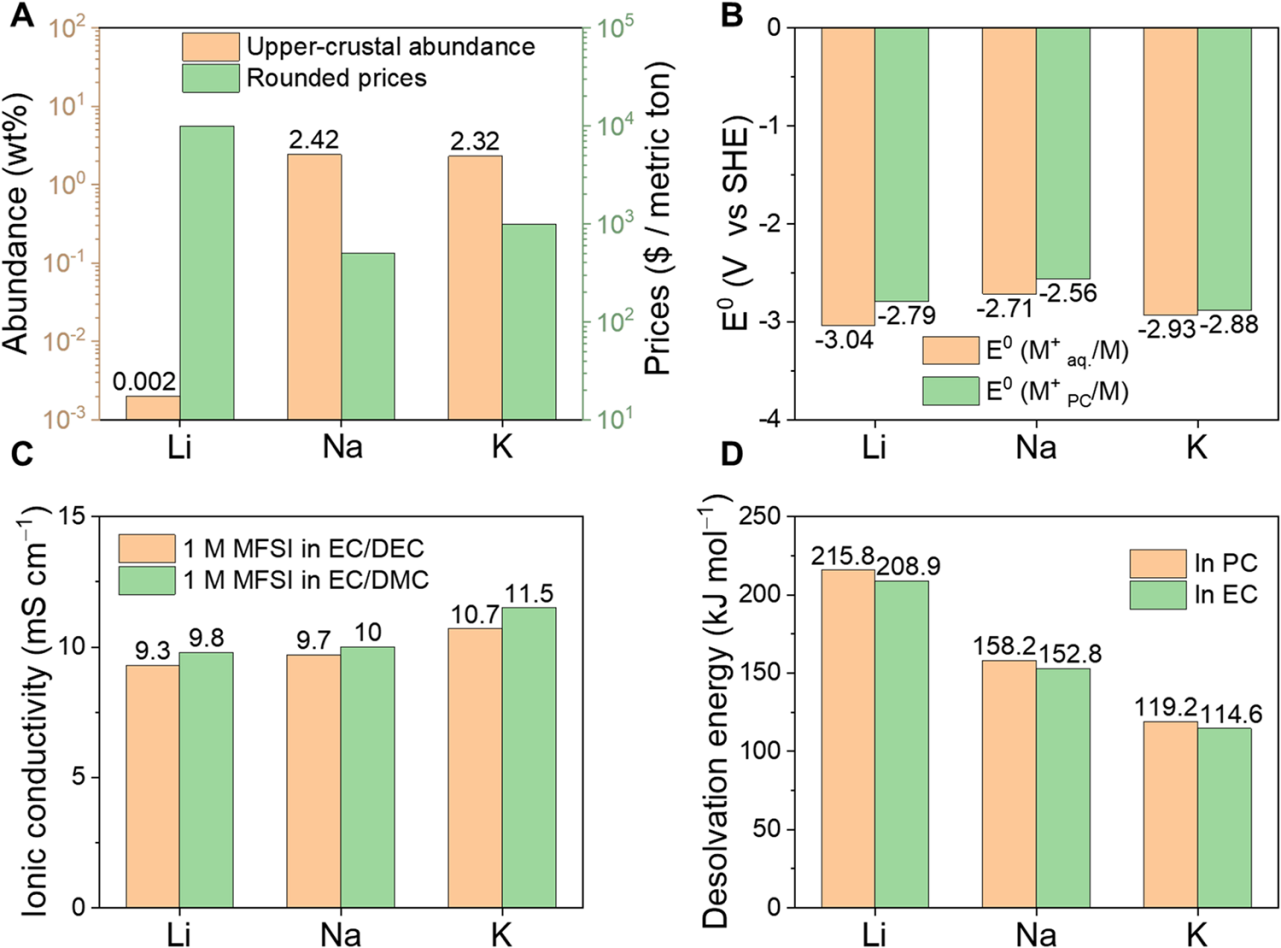
Resource abundances, market prices, and electrochemical properties of Li, Na, and K systems. Aq.—aqueous; M—mol; PC—propylene carbonate; EC—ethylene carbonate; DEC—diethyl carbonate; and DMC—dimethyl carbonate. (A) Upper-crustal abundance and approximate market prices of Li, Na, and K resources. Abundance values are based on recommended compositions of the upper continental crust, where Na and K abundances are derived from Na_2_O and K_2_O values.^7^ Prices represent annual averages defined by USGS standards (2025)^9^ and may fluctuate significantly: Li, contract average of lithium carbonate or lithium hydroxide; Na, average price of soda ash or table salt; K, K_2_O-equivalent or muriate of potash spot average. (B) Standard redox potentials (*E* ^0^) of Li^+^/Li, Na^+^/Na, and K^+^/K couples in aqueous or PC solutions.^18^ (C) Ionic conductivities of Li-, Na-, and K-based electrolytes with carbonate solvents.^19^ (D) Desolvation energies of Li^+^, Na^+^, and K^+^ in electrolytes containing EC or PC solvents.^19^

Electrochemically, the K^+^/K redox couple exhibits a standard potential of −2.93 V versus the standard hydrogen electrode (SHE), lower than that of sodium (−2.71 V for Na^+^/Na), and in propylene carbonate (PC) it is 0.09 and 0.32 V lower than that of Li^+^/Li and Na^+^/Na, respectively (Figure 1B).^18^ These attributes, combined with the high theoretical specific capacity of K metal anode (687 mAh g^–1^), enable high-voltage, energy-dense cell designs. In addition, potassium’s weaker Lewis acidity yields a smaller solvated ion radius than Li or Na, resulting in enhanced ionic conductivity in K electrolytes and lower desolvation energy at the electrolyte/electrode interface (Figure 1C,D).^19^

Unlike Li, K metal is inert to nitrogen, allowing the use of nitrogen atmospheres in battery fabrication in place of costly argon. Furthermore, K does not alloy with aluminum (Al),^20^ enabling the use of low-cost Al (∼1.3 $ per pound) current collectors instead of copper (∼4.2 $ per pound), which further reduces production costs.^9^ Moreover, the low melting point of K metal (63.5 °C, compared to 97.8 °C for Na and 180.5 °C for Li) enables dendrite suppression via Joule heating without igniting inflammable organic solvents, mitigating thermal runaway risks.^21^ Collectively, these advantages position PMBs as a scalable and economically viable solution for large-scale and sustainable energy storage, with the potential to enable more resilient clean energy infrastructures.

Despite the high energy density enabled by the use of K metal anodes, stabilizing K plating/stripping, the half-reaction occurring at the anode, remains a formidable challenge.^22,23^ The pronounced reactivity of K metal readily induces the decomposition of electrolyte solvents and salts upon contact with its surface. This reactivity is further exacerbated during plating, particularly under high-voltage charging conditions,^24^ where an excess of electrons renders the K surface even more reductive. The resulting decomposition products form a passivation layer known as the solid-electrolyte interphase (SEI), which typically consists of an inorganic-rich inner layer and an organic-rich outer layer.^25^ The SEI is critical for preventing continued parasitic reactions between the K metal and the electrolyte, while still allowing K^+^ migration across the interface.

However, due to the “host-less” nature of the K metal anode during plating—where K adatoms directly adsorb onto nucleation sites and integrate into crystalline growth^26^—substantial, often conceptualized “infinite”, volume expansion occurs. Uneven nucleation and self-reinforcing growth also promote dendrite formation and impose severe local mechanical stress on the SEI, causing fracture and exposing fresh K surface.^26^ The renewed surface accelerates electrolyte consumption, exacerbates nonuniform K^+^ flux, and further amplifies dendrite growth.^27^ Under practical conditions—high areal capacity, high current density, limited electrolyte supply, and extreme temperatures—these degradation pathways are intensified, leading to premature failure and severe safety risks. Consequently, controlling K deposition morphology, suppressing dendrite growth, and engineering robust SEI composition and structure are central scientific challenges for advancing PMB technology.^28^

## 2 Strategies for Stabilizing Potassium Metal Anodes

A broad spectrum of stabilization strategies for K metal anodes has been proposed, including electrolyte engineering,^29^ additive regulation,^30^ artificial SEI construction,^31^ separator modification,^32^ and solid-state or polymer electrolytes.^33,34^ These approaches have been comprehensively summarized in existing reviews.^28,35–40^ This article focuses exclusively on substrate design as a distinct yet underexplored strategy for stabilizing K metal anodes. We synthesize recent advances in substrate-engineering approaches that regulate K nucleation, deposition morphology, and SEI formation. Five principal strategies are classified and compared: three-dimensional (3D) conductive hosts, heteroatom doping and molecular grafting, inorganic nanoparticle incorporation, alloying seeds, and work-function engineering. Each strategy is evaluated in terms of its underlying mechanism, demonstrated benefits, practical limitations, and remaining challenges.

### 2.1 Structural Engineering via Three-Dimensional Host Architectures

3D conductive hosts are a primary strategy to mitigate the severe volume changes and dendritic growth of K metal. They (i) expand the effective nucleation area to reduce local current density, (ii) provide internal voids to accommodate deposited K, and (iii) undergo potassiation (e.g., KC_8_ formation) that strengthens K–substrate binding and lowers interfacial impedance. Typically, a 3D host–K composite anode is fabricated via a two-step process: (i) synthesis of a porous, conductive substrate, often carbon-based networks or metallic foams; and (ii) infusion of K metal into the host voids. The morphology of initially deposited K nuclei largely dictates subsequent deposition behavior and, therefore, the cycling stability of the anode.

Applying a simple carbon paper (CP) layer on a K metal surface significantly reduces plating/stripping polarization and improves cycling stability, underscoring the ability of 3D carbon networks to regulate K deposition.^41^ Infusing K metal into carbon nanofiber (CNF)^42^ and carbon nanotube (CNT)^43^ networks further lowers nucleation overpotential and promotes uniform deposition (Figure 2A). This improvement arises from the potassiation of highly graphitic carbon, forming KC_8_ prior to plating, which decreases interfacial impedance compared to bare K metal foil (Figure 2B). This KC_8_ phase increases the binding energy of K atoms from −0.78 eV for graphite (110) and −0.73 eV for K (110) to −1.55 eV for KC_8_ (002), thereby enhancing the potassiophilicity of the carbon host.^44^ This, combined with reduced local current density, contributes to the observed decrease in nucleation overpotential and impedance.

**Figure 2:**
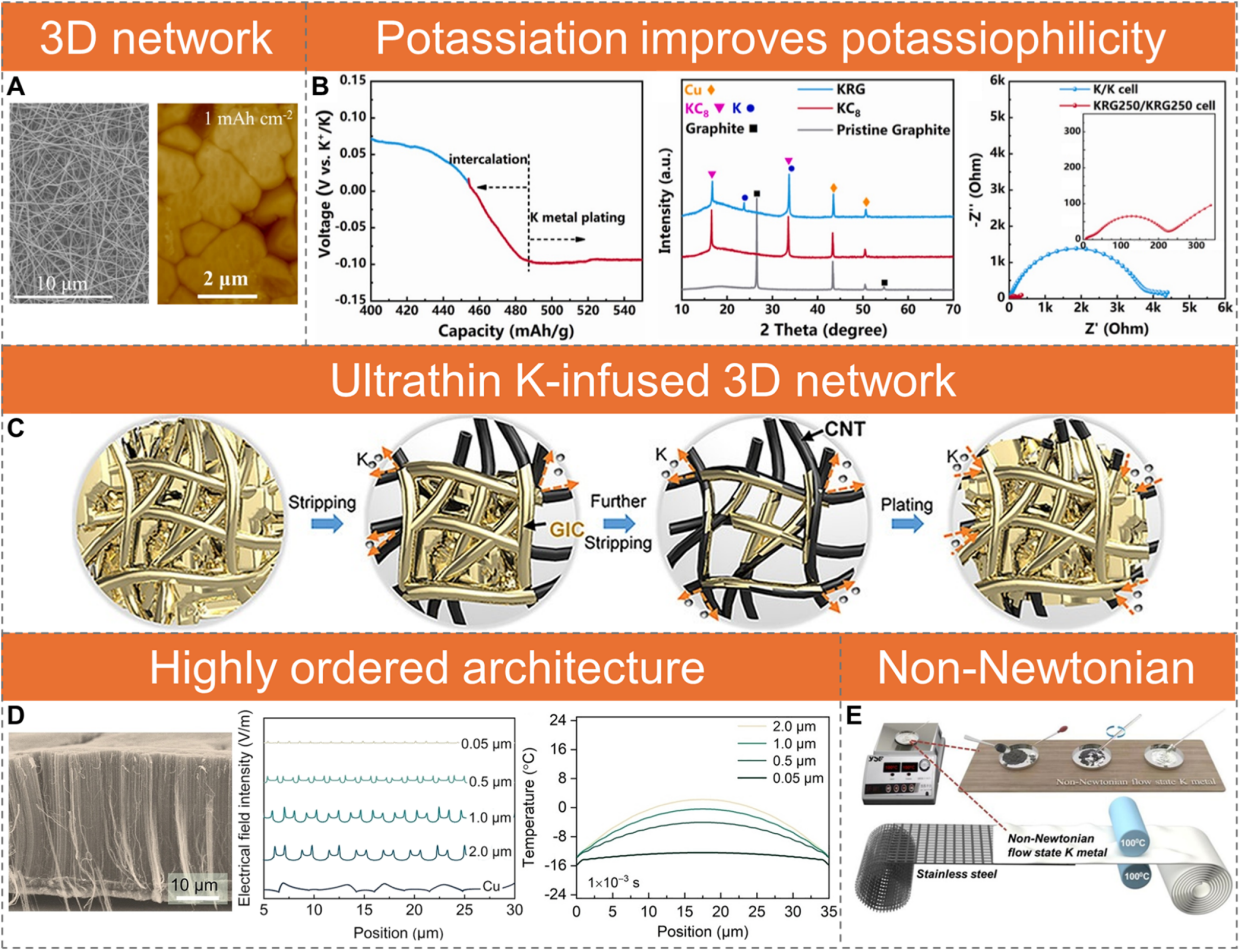
Structural engineering strategies for 3D host architectures. (A) SEM image of CNF and AFM image of K deposition (1 mAh cm^–2^) on CNF. Reproduced with permission from ref 42. Copyright 2022 Elsevier. (B) Galvanostatic voltage profiles of K||graphite cells showing K intercalation and plating at a current density of 25 mA g^–1^; XRD patterns of graphite anodes indicating the formation of KC_8_ and K-rich graphite (KRG) during discharge; and Nyquist plots of K||K and KRG||KRG symmetric cells. Reproduced with permission from ref 44. Copyright 2023 Elsevier. (C) Schematic illustration of the K plating/stripping process within CNT networks. Reproduced from ref 45. Copyright 2024 American Chemical Society. (D) Cross-sectional SEM image of highly ordered CNTs, showing a dense film structure; electric field distribution plotted along the electrode, revealing high uniformity in regions with interspace gaps below 0.05 μm; temperature distribution of the thermal field at a highly ordered 1D nanoarray electrode with varying gap widths at 1 × 10^–3^ s. Reproduced with permission from ref 48. Copyright 2023 John Wiley and Sons. (E) Schematic of the synthesis process for non-Newtonian flow-state K metal using a stainless-steel substrate. Reproduced with permission from ref 50. Copyright 2023 Elsevier.

CNTs, with a high specific surface area of 92.2 m^2^ g^–1^, can increase the K binding energy up to −3.09 eV.^45^ The strong interaction within the K@CNT composite significantly enhances its mechanical properties, increasing the Young’s modulus from 1.4 GPa (K metal) to ∼8.8 GPa and elevating the effective melting point from 63.5 to ∼300 °C. This enables fabrication of flexible electrodes with tunable thicknesses ranging from ∼30 to ∼200 μm and corresponding areal capacities from ∼1.77 to ∼11.82 mAh cm^–2^. The K@CNT composite anode enables uniform K plating/stripping along the conductive CNT network, effectively suppressing dendrite growth via in-plane ion regulation (Figure 2C). It supports high areal current densities up to 10 mA cm^–2^ with stable cycling in symmetric cells. In full cells with a Prussian white (PW) cathode (∼7.7 mg cm^–2^), it delivers a reversible capacity of 65.8 mAh g^–1^ at 200 mA g^–1^. A 12 mAh pouch cell further achieves 187.3 Wh kg^–1^ without prepotassiation, demonstrating the viability of K-infused CNTs as scalable alternatives to bare K metal anodes.

Aligned CNT architecture further enhances the electronic and ionic transport, improving redox kinetics and promoting stable cycling and capacity retention in PMBs.^46,47^ Highly ordered one-dimensional (1D) CNT nanoarrays with sub-0.05 μm spacing ensure uniform electric and thermal field distributions (Figure 2D).^48^ K deposition on these aligned CNTs results in large, smooth domains, in stark contrast to the irregular, whisker-like, or fractured deposits observed on graphene and Cu electrodes. Notably, highly ordered CNTs enable stable K plating/stripping at −20 °C for over 70 cycles, whereas Cu and graphene electrodes fail under the same conditions. Full cells using perylene-3,4,9,10-tetracarboxylic dianhydride (PTCDA) cathodes retain over 80% of their room-temperature capacity at −20 °C, further demonstrating the low-temperature viability of this system. These findings underscore the critical role of electric field regulation for uniform deposition.^49^

Moreover, mixing K metal with carbon black (Super P) at 80 °C produces a non-Newtonian K@Super P composite exhibiting shear-thinning and thixotropic behavior, likely due to bond formation between K and Super P (Figure 2E). When coated onto stainless steel, this composite forms a self-supporting, flexible electrode that reduces localized stress and prevents cracking.^50^

3D hosts effectively reduce nucleation overpotential, suppress dendrite formation, and enable high areal capacity and improved mechanical resilience. However, many high-surface-area architectures compromise tap/volumetric density, increase electrolyte uptake, and introduce fabrication complexity, making lean-electrolyte operation difficult. Moreover, spatially resolved deposition pathways within complex pores remain insufficiently understood. Future work should (i) optimize pore structure and surface area to balance areal and volumetric energy density, (ii) establish scalable, low-cost synthesis of ordered 3D hosts, and (iii) integrate operando imaging with modeling to elucidate deposition dynamics under practical conditions such as high areal loading, lean electrolyte, and varied temperatures.

### 2.2 Electron Localization via Heteroatom Doping and Molecular Grafting

Heteroatom doping and molecular grafting modify local electronic structures and create dipolar or charged sites that significantly enhance K adsorption energy and wettability, thereby guiding nucleation and promoting uniform deposition. First-principles calculations on graphene nanoribbons doped with B, N, O, F, P, S, Cl, Br, and I reveal a wide range of binding energies for K atoms (Figure 3A).^51^ Among these, F, Cl, Br, and I are identified as ineffective dopants, exhibiting lower K binding energies than pristine graphene. In contrast, effective dopants such as B–2C–O-type boron (oB) codoping exhibit the highest binding energy (−2.86 eV), followed by P-doping (−2.67 eV), carboxyl groups (aO), sulfonyl groups (oS), and pyridinic nitrogen (pN). These high binding energies are attributed to strong dipoles formed between the doping atoms and their adjacent atoms, as well as significant charge transfer (Bader charge) exceeding 0.89 electrons from K atoms to the doping sites (Figure 3A).

**Figure 3:**
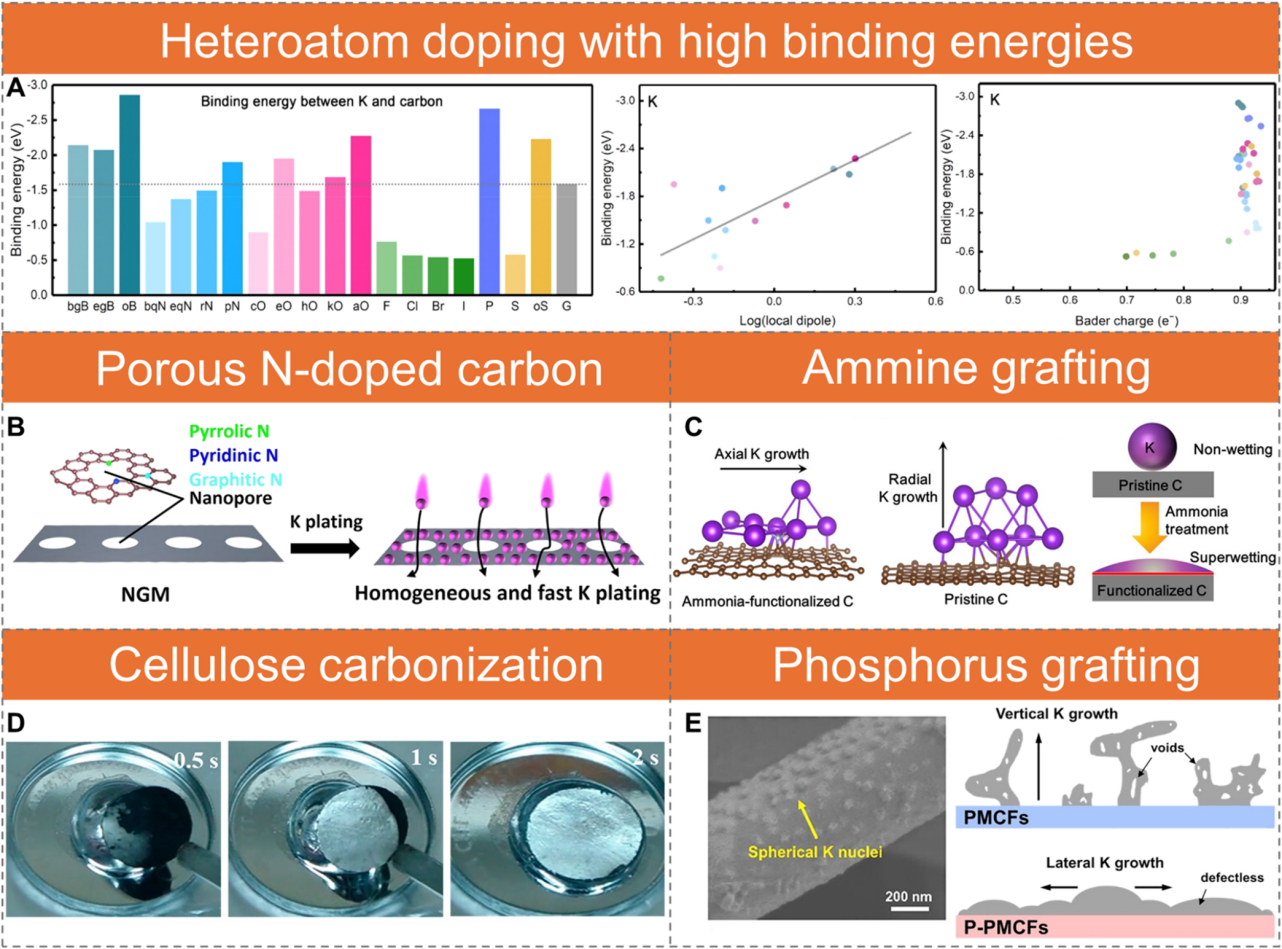
Heteroatom doping and molecular grafting strategies for electron localization. (A) Binding energy between K atoms and doped carbons, showing the correlation between K binding energy, log­(local dipole), and charge transfer. Reproduced with permission from ref 51. Copyright 2020 Elsevier. (B) Schematic of a porous N-doped carbon that enables homogeneous and rapid K plating. Nanopores provide direct pathways for K ions to access the entire electrode. Reproduced with permission from ref 52. Copyright 2022 Elsevier. (C) DFT calculations of K growth on ammonia-treated and untreated carbon surfaces. Atom colors: C (gray), N (blue), H (white), K (purple). Inset: schematic illustration showing significantly improved wettability of K metal on the functionalized carbon host. Reproduced from ref 53. Copyright 2022 American Chemical Society. (D) Photographs showing molten K infusion into carbonized bacterial cellulose scaffolds, demonstrating high potassiophilicity. Reproduced from ref 54. Copyright 2021 American Chemical Society. (E) SEM images of K nucleation morphology on P-grafted porous CNFs after K plating at 1 mA cm^–2^ for 18 s, along with schematics of K growth behavior on porous CNFs and P-grafted porous CNFs. Reproduced from ref 62. Copyright 2024 American Chemical Society.

Experimental evidence supports these predictions. Pyridinic N doping reduces charge transfer resistance and polarization, while micro/nanoporous structures in N-doped carbon facilitates rapid K^+^ transport, yielding a high K^+^ diffusion coefficient (Figure 3B).^52^ Similarly, NH_3_-functionalized carbon cloth (CC) exhibits strong wettability toward molten K, guiding axial deposition and preventing dendritic growth, unlike pristine CC, which remains nonwetting and induces radical K growth (Figure 3C).^53^ Carbonization of bacterial cellulose produces potassiophilic CNFs enriched with oxygen-containing functional groups, such as carboxyl and ketone groups, which exhibit strong K adsorption and high potassiophilicity with rapid molten K infusion (Figure 3D), thereby lowering the K nucleation overpotential.^54^ Oxygen functionalities can also be introduced through reduced graphene oxide (rGO),^55–57^ molecular oxygen grafting,^58^ air heat treatment,^59^ or annealing with oxygen-rich precursors.^60^ Codoping with N, O, and S further enhances performance.^61^ Additionally, porous CNFs grafted with red phosphorus via evaporation-deposition form P nanoclusters anchored by C–P bonds, creating abundant K nucleation sites.^62^ This design promotes uniform in-plane deposition and suppresses perpendicular dendrite growth, resulting in 85% capacity retention after 1000 cycles at 20 C for perylene-3,4,9,10-tetracarboxylic dianhydride (PTCDA) cells (Figure 3E).

These approaches offer atomic-level tunability, compatibility with low-cost carbon scaffolds, and consistent reductions in nucleation barriers, leading to improved deposition uniformity. Their challenges include identifying the most effective dopant motifs (type, site, and coverage), mitigating the instability of grafted groups during cycling, and ensuring reproducibility at scale. Future research should integrate targeted DFT screening with standardized operando diagnostics to establish structure–function correlations, while prioritizing designs that preserve dopant functionality under repeated cycling and lean-electrolyte conditions.

### 2.3 Electron Localization via Inorganic Incorporation

Decorating hosts with inorganic nanoparticles or embedding active phases induces local electron redistribution and generates strong adsorption sites for K, thereby lowering nucleation overpotential and improving deposition morphology. Transition metals such as Ag,^63,64^ Au,^65^ and Pd,^66^ as well as alloys like Cu_6_Sn_5_,^67^ Cu_3_Pt,^68^ and GaInNi,^69^ exhibit strong binding energies with K, effectively lowering nucleation overpotential and promoting uniform K deposition. High-entropy alloys (HEAs), composed of five or more metallic elements, generate surface regions with electron accumulation and depletion, thereby enhancing K^+^ adsorption. For instance, equimolar MnFeCoCuNi HEA nanoparticles exhibit electron enrichment at Cu and Ni sites, enhancing K^+^ binding beyond that of the corresponding elemental metals (Figure 4A).^70^ This enables PTCDA cells to deliver a reversible capacity of 66 mAh g^–1^ (58% capacity retention) and nearly 100% Coulombic efficiency (CE) after 2000 cycles at 20 C.

**Figure 4:**
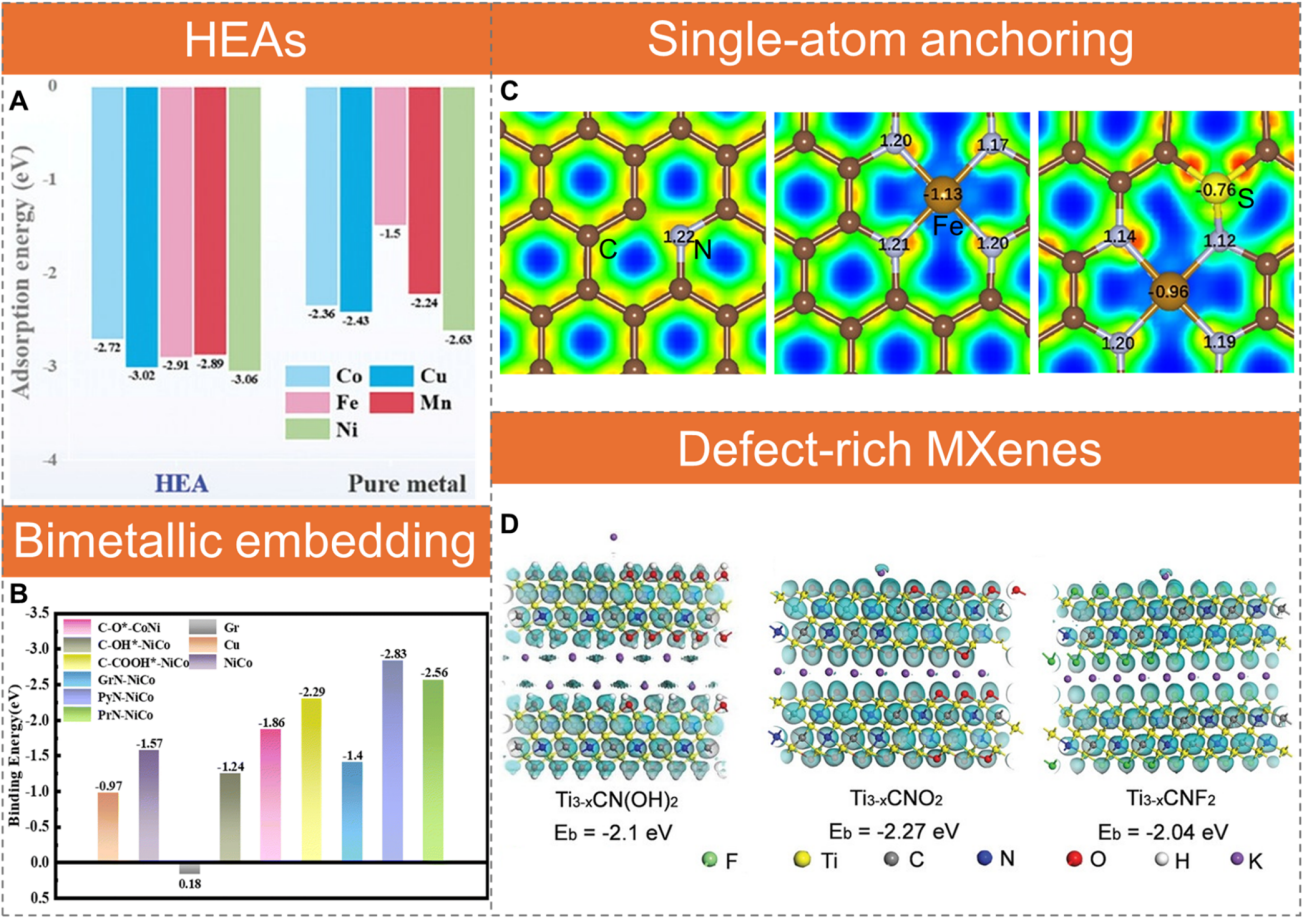
Inorganic incorporation for electron localization. (A) Adsorption energies of a K ion on Mn, Fe, Co, Cu, and Ni sites in HEA compared to their pure metal counterparts. Reproduced with permission from ref 70. Copyright 2024 John Wiley and Sons. (B) Binding energies of a K atom on Cu and on pristine and modified NiCo@N,O-doped graphene surfaces. Reproduced with permission from ref 72. Copyright 2023 Royal Society of Chemistry. (C) Electron localization function (ELF) maps of N-doped graphene, single-atom Fe@N-doped graphene, and single-atom Fe@N,S-doped graphene. Electron density increases from blue to green, yellow, and red. Reproduced with permission from ref 76. Copyright 2025 John Wiley and Sons. (D) Density functional theory (DFT)-calculated deformation charge density of K atoms adsorbed on Ti_3–x_CNO_2_, Ti_3–*x*_CN­(OH)_2_, and Ti_3–*x*_CNF_2_. Reproduced with permission from ref 85. Copyright 2019 John Wiley and Sons.

Synergistic effects also arise when metals are embedded into heteroatom-doped carbon. For example, elemental Co alone binds weakly with K^+^, but when embedded in N-doped carbon, it increases surface electron density of the carbon, enhancing K^+^ adsorption and improving potassiophilicity.^71^ Similar benefits are observed in bimetallic systems such as NiCo^72^ and CoZn,^73^ where NiCo embedded in N,O-doped carbon exhibits the strongest K atom adsorption at the pyridinic N configuration, surpassing both O-containing carbon@NiCo and bare NiCo (Figure 4B). Downsizing metals to quantum dots or single atoms further amplifies their effectiveness. Cu quantum dots,^74^ single-atom Co,^75^ and single-atom Fe^22^ anchored on N-doped or N,S-doped carbon provide abundant nucleation sites and exhibit strong K adsorption, driven by enhanced electron localization at N sites induced by the metal nanoclusters or atoms.

Single-atom Fe, coordinated with N dopants (Fe–N_4_), increases electron density at adjacent N sites, while additional S doping further strengthens electron localization (Figure 4C).^76^ The integration of Fe nanoclusters with single-atom Fe promotes cooperative electron donation, shifting the d-band center closer to the Fermi level and thereby strengthening overall K binding.^77^ Compounds with localized electrons can also show strong K binding; examples include CoWO_4_ (electron localization via lattice distortion)^78,79^ and α-phase MoC (significant Mo-to-C electron transfer).^80,81^

MXenes, a family of two-dimensional (2D) transition metal carbides, nitrides, and carbonitrides, form strong covalent metal (M)-C/N bonds and can promote uniform deposition.^82,83^ Ti_3_C_2_ MXene nanoribbons, for instance, provide a 3D woven-like framework for uniform K plating.^84^ Introducing Ti vacancies and surface terminations (−O, −OH, −F) in Ti_3–*x*_CNT_*y*_ structures further improves K binding energy and reduces nucleation overpotential from 33 mV to as low as 6 mV.^85^ The surface groups modulate the electronic structure of adjacent C/N atoms,^86^ yielding binding energies of −2.27 eV for Ti_3–*x*_CNO_2_, −2.10 eV for Ti_3–*x*_CN­(OH)_2_, and −2.04 eV for Ti_3–*x*_CNF_2_ (Figure 4D).^85^ Zeolitic imidazolate framework-8 (ZIF-8), a nitrogen-rich metal–organic framework (MOF), also enhances plating/stripping stability through its nanoporous structure, abundant surface functional groups, and nitrogen active sites.^87^

Inorganic additives provide strong and tunable K binding, reinforce host mechanics, and create synergistic effects, such as enhanced electron density and cooperative binding, when integrated with doped carbons. However, challenges include heterogeneous dispersion, potential electronic insulation if poorly integrated, unintended catalytic side reactions with electrolytes, high costs for certain metals, and scale-up difficulties. The long-term electrochemical stability of nanoparticle–electrolyte interfaces also requires rigorous evaluation. Future efforts should focus on engineering well-integrated, conductive inorganic–carbon interfaces with controlled spatial distribution, emphasizing earth-abundant elements, conductive encapsulation to avoid insulating shells, and systematic operando/accelerated aging studies.

### 2.4 Interfacial Reconstruction via Alloying Seeds

Alloying seeds provide a different approach: instead of passive adsorption, reactive seeds form K-containing alloys that act as thermodynamically favorable nucleation sites with near-zero interfacial energy, thereby reducing nucleation overpotential and enabling homogeneous deposition.^26^ When dispersed within a 3D matrix, these alloying seeds undergo spontaneous alloying reactions, which are thermodynamically favorable, and subsequently promote site-specific deposition on the resulting alloy phases. To date, Bi-, Zn-, and Sn-based metals or their reactive compounds have been most widely explored.

Bi-based alloying seeds have been implemented via Bi nanoparticles,^88,89^ direct surface coatings,^90^ and reactive oxides such as Bi_2_O_3_.^91^ For instance, Bi_80_ nanoclusters anchored on N-doped rGO develop dense hollow pores after molten K infusion (Figure 5A).^92^ The dynamic alloying-dealloying process enables reversible pore restoration during cycling, directing K to deposit within the pores, suppressing dendrites, and lowering the nucleation overpotential to ∼5 mV. The PW cells achieve a capacity of ∼65 mAh g^–1^ with negligible degradation and ∼99% CE at 1000 mA g^–1^ after 1960 cycles.

**Figure 5:**
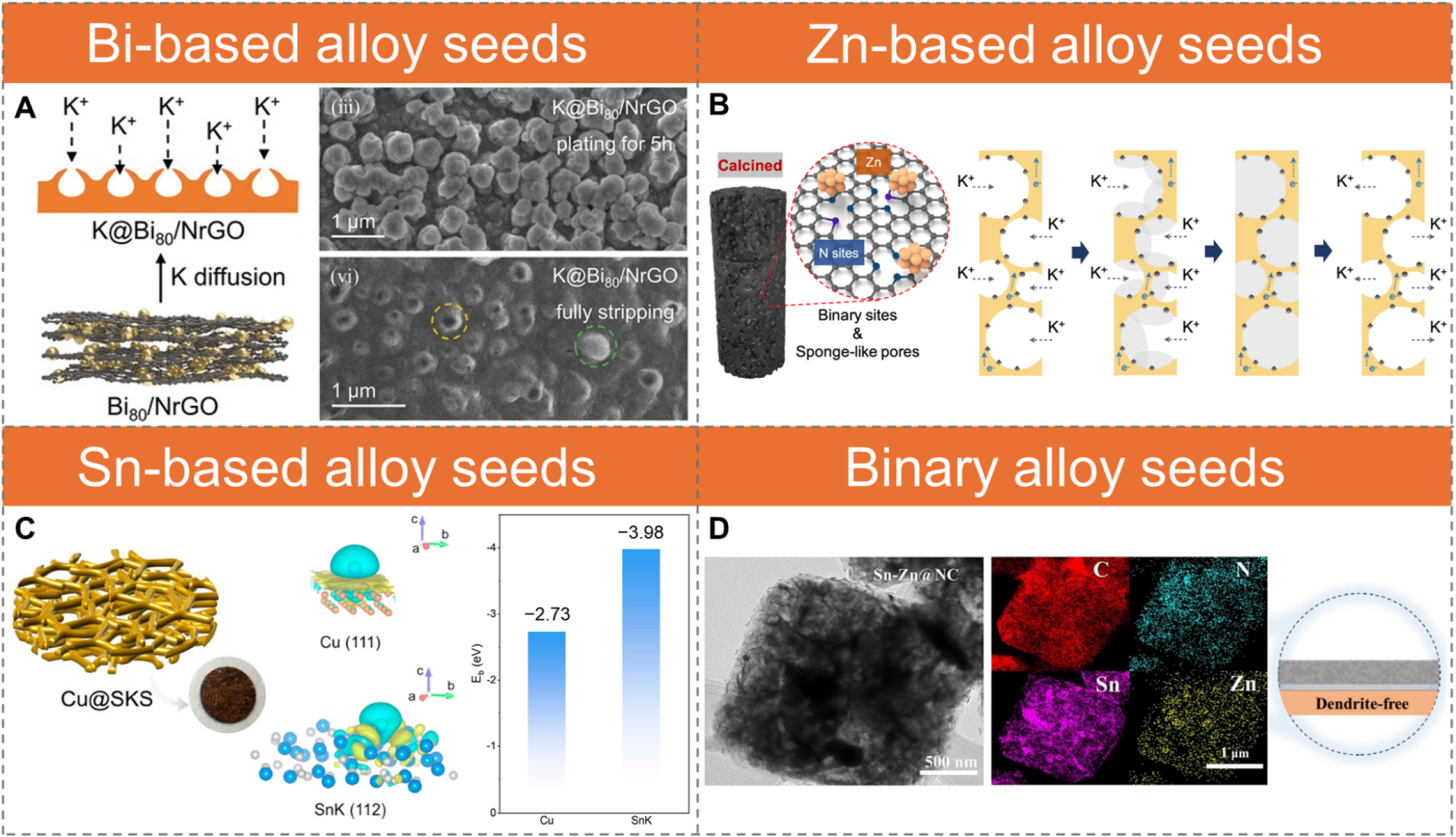
Alloying-seed strategies for interfacial reconstruction. (A) Schematic illustration of K plating on a K@Bi_80_ anchored on N-doped rGO. SEM images show the evolution of K morphology during plating/stripping. During plating, the hollow pores of K@Bi_80_ gradually fill with K metal, forming small, brighter spheres. After stripping, the hollow pore structure reappears with lower contrast (yellow dashed circles), indicating structural preservation and residual unstripped K. Reproduced with permission from ref 92. Copyright 2023 John Wiley and Sons. (B) Schematic of N-doped porous CNFs anchored with Zn clusters and the corresponding K plating/stripping process. Reproduced from ref 99. Available under a CC-BY 4.0 license. Copyright 2022 Siwu Li et al. (C) Schematic and optical image of the SnK alloy@Cu substrate. Differential charge density plots at K^+^ adsorption sites on Cu(111) and SnK(112) surfaces. Yellow and light blue indicate charge accumulation and depletion, respectively (isosurface level: 0.0002 e Bohr^–3^). Blue, gray, and orange spheres represent K, Sn, and Cu atoms. Binding energies of Cu and SnK alloy are also shown. Reproduced from ref 105. Copyright 2023 American Chemical Society. (D) TEM image of binary Sn–Zn@N-doped carbon and corresponding elemental mappings of C, N, Sn, and Zn. Schematic illustration of uniform K deposition. Reproduced with permission from ref 106. Copyright 2025 Elsevier.

Zn-based alloying seeds, including metallic Zn^93^ and compounds such as ZnO,^94–96^ ZnF_2_,^97^ and ZnTe,^98^ react with K to form Zn–K alloys during plating. Zn nanoclusters embedded in porous N-doped CNFs generate dual active sites: Zn functions as the alloying center, while N dopants provide strong K binding (Figure 5B).^99^ The uniform distribution of these binary seeds within high-surface-area nanopores promotes homogeneous K nucleation and deposition, thereby enhancing deposition uniformity and improving host volume utilization (Figure 5B).

Sn-based alloying seeds, derived from elemental Sn,^100^ SnO_2_,^101–103^ SnS_2_,^104^ or SnBr_2_,^25^ also show strong potassiophilicity. Sn-coated Cu foam, after prepotassiation, forms SnK alloys with a high K binding energy (−3.98 eV for SnK (211) versus −2.73 eV for Cu(111)), enabling stable plating/stripping (Figure 5C).^105^

Binary alloying seeds are further investigated. Comparative studies indicate that binary Sn–Zn outperforms binary Bi–Zn in cycling stability, likely due to lower K^+^ diffusion barriers in KSn_2_ (Figure 5D).^106^ Other alloying elements such as Sb,^107^ Sb_2_O_3_,^108^ GeO_2_,^109^ and Hg^110^ have also proved effective in extending PMB cycling life. In addition to alloy seeds, certain reactive but nonalloy-forming compounds—such as partially selenized copper oxyselenide (Cu–OSe) nanowires,^111^ CuSe coatings,^112^ MoS_2_ microparticles,^113^ CuO nanoparticles,^114^ nanomesh porous NiO,^115^ NiO nanoparticles,^116^ and S layers^117^—have demonstrated improvements in cycle stability by modifying deposition pathways and suppressing dendrite growth.

Alloy seeds achieve extremely low nucleation barriers and dynamically reconstruct interfaces to confine deposition within engineered pores, yielding excellent morphological control and stable cycling. Their limitations include side reactions, partial irreversibility of alloying–dealloying, volume fluctuations in alloy phases, and possible dependence on costly or toxic elements, all of which challenge long-term stability and scalability. Future directions include (i) designing alloy compositions that balance potassiophilicity, reversibility, and cost, (ii) developing confinement strategies to prevent seed agglomeration or pulverization, and (iii) conducting mechanistic studies to quantify alloying kinetics and their interplay with SEI evolution under practical cycling conditions.

### 2.5 SEI Engineering via Substrate Work Function

The substrate not only governs the morphology of K deposition but also shapes the composition and structure of the SEI. One straightforward approach involves pretreating substrates with SEI-forming compounds (e.g., NaF) to promote robust, inorganic-rich interphases.^118^ Substrate electronic properties, particularly its Fermi level (*E*_f_) and work function (Φ = *E*_vac_ – *E*_f_, where *E*_vac_ = 0 eV), directly affect interfacial electron flux during early plating, thereby determining electrolyte decomposition pathways and the organic/inorganic balance of the SEI.

A higher work function (lower *E*_f_) decreases electron transfer to electrolyte molecules, suppressing the decomposition of neutral solvents while allowing preferential anion decomposition, thereby producing inorganic-rich SEIs. For example, direct growth of N-doped graphene on porous Al (NG@P–Al) via plasma-enhanced chemical vapor deposition raises the work function from 2.88 eV (bare Al) to 3.97 eV (Figure 6A), as measured by ultraviolet photoelectron spectroscopy (UPS).^119^ After K plating and stripping, NG@P–Al retains a high work function of 3.76 eV, compared with 2.67 eV for bare Al. The resulting SEI contains the lowest fraction of organic species of poly­(CO_3_), C–F, and N_*x*_O_*y*_ species, and the highest KF content, indicating a high inorganic-to-organic ratio and enhanced salt-derived composition.

**Figure 6:**
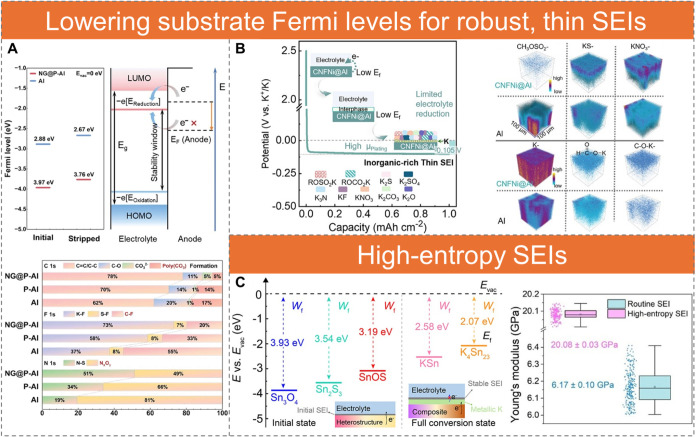
Substrate work function modulation for SEI engineering. (A) Fermi levels of NG@P–Al and Al at the initial and stripped stages, determined by extrapolating the secondary cutoff region in the UPS profiles. Schematic of SEI formation at the anode interface, and SEI chemical configurations with corresponding content percentages for all current collectors at the formation stage. Reproduced with permission from ref 119. Copyright 2023 John Wiley and Sons. (B) Schematic of SEI formation on CNFNi@Al current collectors during the first K plating in a three-electrode system without a formation period. TOF-SIMS 3D renderings of CH_3_OSO_2_, KS, KNO_3_, K, HCO_2_K, and C–O–K signals. Reproduced with permission from ref 120. Copyright 2025 John Wiley and Sons. (C) Calculated work functions of Sn_3_O_4_, Sn_2_S_3_, SnOS heterostructure, KSn, and K_4_Sn_23_, along with statistical values of Young’s modulus. Reproduced with permission from ref 122. Copyright 2025 John Wiley and Sons.

Similar effects are achieved by directly spraying nickel (Ni)-embedded carbon nanofibers (CNFNi@Al) onto Al current collectors.^120^ The embedded Ni species (Ni, NiO, Ni_3_N, and Ni_3_C) and carbon framework increase the work function to 4.2 eV, attributed to the higher electronegativity of Ni and CNF compared with Al, which reduces electron density at the surface and limits solvent decomposition (Figure 6B). This promotes the formation of a thin SEI and lowers electron tunneling probability, as the decreased E_f_ suppresses electron tunneling in quantum mechanical terms. CNFNi@Al also lowers nucleation overpotential and raises the plating plateau to −0.105 from −0.152 V for Al, which intensifies electrolyte decomposition and yields an organic-rich SEI. Time-of-flight secondary ion mass spectrometry (TOF-SIMS) confirms that CNFNi@Al produces a thin, uniform SEI enriched in anion-derived species (KS^–^, KNO_3_^–^) with minimal solvent decomposition products (CH_3_OSO_2_^–^), whereas bare Al exhibits deeper solvent degradation, producing HCO_2_K-dominated layers throughout the SEI (Figure 6B). Under a low N/P ratio of 1, the PW cells deliver ∼105 mAh g^–1^ at 100 mA g^–1^ for 100 cycles. The low-Fermi-level current collector strategy has also proven effective in LMBs.^121^

Borrowing from the concept of high-entropy materials, a “high-entropy SEI” strategy has been proposed, incorporating five or more inorganic components to generate abundant grain boundaries that facilitate K^+^ transport and reduce concentration gradients.^122^ For example, hydrothermally coating 3D nickel foam with a Sn_3_O_4_/Sn_2_S_3_ heterostructure (SnOS@NF) induces complex electrochemical reactions that yield K_2_SO_3_, K_2_CO_3_, K_2_S, K_2_O, and KF during cycling. After complete conversion during K plating, the heterostructure transforms into KSn and K_4_Sn_23_ alloys with low work function values of 2.58 and 2.07 eV, respectively, significantly lower than those of Sn_3_O_4_, Sn_2_S_3_, or the SnOS heterostructure (Figure 6C). This leads to further electrolyte decomposition to enrich the SEI components. The diversified components produce a high-modulus SEI (average ∼ 20 GPa) compared with conventional SEIs (∼6 GPa), thereby enhancing mechanical integrity. This allows the PW cells to retain 82.9 mAh g^–1^ after 1050 cycles at 2C.

Looking forward, integrating substrate engineering with controlled sacrificial chemistries offers a promising pathway: (i) tailoring or grading substrate work functions to homogenize electron flux, and (ii) seeding multicomponent inorganic SEIs for enhanced stability. Future efforts should focus on mapping work function (Kelvin probe force microscopy,^123^ scanning tunneling spectroscopy^124^) and SEI composition (TOF-SIMS), quantifying SEI entropy and mechanics, and leveraging data-driven screening of precursor combinations. Scalable fabrication methods such as spray coating, plasma-enhanced chemical vapor deposition (PECVD), or atomic layer deposition (ALD) will be key for practical translation, with attention to compatibility under lean electrolyte and high areal capacity conditions. Key risks to manage are interfacial impedance from overly insulating layers and unintended catalytic routes from embedded metals.

## 3 Conclusions and Outlooks

Stabilizing K metal anodes requires a multifaceted strategy that integrates structural, chemical, and electronic design principles. Progress in understanding K deposition behavior and SEI formation, combined with scalable and cost-effective fabrication methods, is essential for advancing PMBs as sustainable, high-performance energy storage technologies. Substrate design provides promising pathways, and Table 1 summarizes the advantages and limitations of current strategies, highlighting mechanistic insights and unresolved challenges.

**Table 1:** Comparison of Various Substrate Design Strategies on Their Primary Mechanisms, Advantages, Challenges, Material Cost, Synthesis Complexity, and Near-Term Scalability^a^

strategy	representative materials	primary mechanisms	advantages	challenges	material cost (1–5)	synthesis complexity (1–5)	near-term scalability (1–5)
3D architectures	Carbon foams, CNT/CNF networks, MXene aerogels, metal foams	Lower local current density; buffer volume changes; guide uniform nucleation	Compatible with roll-to-roll; strong mechanical tolerance; dendrite suppression	Dead K accumulation if pores not wetted; molten K infusion often required	2–4	3–4	3
Doping and grafting	N, O, S (co)doped carbons; P and NH_3_ grafted carbons	Enhance binding energy via electron localization; provide nucleation sites	Reduce nucleation overpotential; uniform K deposition	Rely on 3D carbon hosts; doping level control and reproducibility remain issues	1–2	2–3	4
Inorganic incorporation	Transition metals, alloys, carbides, oxides, nitrides, single-atom, MXenes	Enhance binding energy via electron localization; provide nucleation sites; inorganic-rich SEI	Reduce nucleation overpotential; strong chemical anchoring	Rely on 3D hosts; interfacial stability concerns and brittleness; complex synthesis for some materials	3–5	3–4	2–3
Alloying seeds	Bi, Zn, Sn, Sb, and Ge thin layers or particles	Transient K-M alloying lowers K nucleation barrier; abundant nucleation sites	Low nucleation overpotential; selective K plating	Use 3D hosts to improve efficiency; large volume fluctuations of alloying and dealloying	3–4	3–5	2
Substrate work function	N-doped graphene layers, Ni-embedded CNFs; Sn_3_O_4_/Sn_2_S_3_ heterostructures	Work function control preforms inorganic-rich, thin, ion-conductive, and robust SEIs	Lower continuous SEI repair, improved CE; manufacturing-friendly	Adhesion and thickness control are critical; work function control worths further study	2–3	3	4

a Semi-quantitative evaluation scores 1–5 from low to high.

From a commercialization perspective, scalability and cost remain decisive. 3D carbon-based hosts, heteroatom-doped carbons, and carbons incorporated with inorganic components deliver strong performance at relatively low cost, particularly when derived from biomass or inexpensive precursors. Alloying-seed strategies are highly effective in controlling deposition but often rely on costly elements. Substrate work function engineering offers a scalable, manufacturing-compatible route, while high-entropy SEI designs show potential for long-term stability but require careful precursor selection.

Although significant progress has been made, the next decisive step is translating proof-of-concept advances into scalable and manufacturable technologies. This transition can be accelerated by the following priorities, supported by targeted experimental efforts.

### 3.1 K Deposition Pathways in 3D Hosts

3D hosts are among the most promising strategies for stabilizing K metal anodes, yet the spatial pathways of K deposition within these architectures remain poorly resolved. K can deposit preferentially at the top surface, within the bulk, or at the bottom of the host, and the dominant pathway critically determines host volume utilization, mechanical stability, and interfacial uniformity. To optimize host architectures, it is essential to establish how structural parameters (pore size, tortuosity, and conductivity gradients), chemical functionalities (heteroatom doping and inorganic incorporation), and electronic properties influence deposition pathways. These mechanistic insights will guide the rational design of 3D hosts with high K utilization, minimal dead volume, and long-term stability.

### 3.2 Substrate–Electrolyte Synergy

Substrate design cannot be optimized in isolation from electrolyte development. Electrolytes dictate interfacial reactions, SEI composition, and ionic transport, and their properties can either reinforce or offset the advantages of advanced substrates. For example, fluorinated solvents^125^ and weak-solvation electrolytes^126^ often yield inorganic-rich SEIs that synergize with substrates engineered for strong potassiophilicity, while functional additives^127^ help stabilize interfaces during repeated plating and stripping.

The SEI on alkali-metal anodes is a dynamic, multilayered, and spatially heterogeneous film whose nanoscale morphology and chemistry evolve during cycling and with changes in electrolyte solvation.^128,129^ Cryo-TEM and correlative chemical imaging have directly visualized nanoscale stratification, trapped metallic inclusions, and the nucleation of “dead” metal, correlating these features with local overpotential buildup and macroscopic electrochemical degradation.^130^

Inorganic fluorides such as LiF preferentially accumulate in the inner, inorganic-rich SEI sublayer during cycling.^131^ While an LiF-rich layer enhances mechanical robustness, it may also hinder Li^+^ transport under certain conditions, leading to ion-transport limitations.^132,133^ Compared with Li, Na and K generally form more fragile and dynamically reconstructed SEIs and suffer greater initial losses of active metal inventory.^134^ Consequently, reducing the intrinsic reactivity of solvent and salt components^135^ represents a key design principle for improving interfacial stability in these systems.

Future progress will depend on codesign strategies that integrate substrate architecture and electrolyte formulation to establish robust, self-adaptive SEIs. Such synergistic approaches are likely to offer the most promising pathway toward durable, high-performance PMBs.

### 3.3 Integrated Data-Driven Modeling and Mechanistic Characterization

Couple atomistic, mesoscale, and continuum models with experimental data to build predictive frameworks for dendrite formation,^136^ interfacial evolution,^137^ and long-term cycling.^138^ Multiscale modeling that couples DFT with phase-field simulations and continuum-scale models will be essential to capture K nucleation kinetics, ion transport, and stress evolution within complex 3D architectures. Employ machine learning for electrolyte and interface screening with uncertainty quantification to guide experimental design.^139^

Standardized post-mortem analyses of both electrodes should be performed to link degradation to performance decay. Key techniques include time-resolved operando microscopy and spectroscopy,^140^ cryo-TEM for intact interface imaging,^141^ operando XPS^142^ and ToF-SIMS^143^ for surface chemistry, neutron scattering^144^ and solid-state NMR^145^ for bulk and buried phases, and coupled gas analysis^146^ with impedance monitoring to reveal failure pathways.^147^

### 3.4 Integration with Full-Cell Configurations and Scale-Up

Advance to full cells pairing high-performance cathodes with optimized electrolytes and realistic negative-to-positive capacity (N/P) ratios. Validate performance in long-format pouch cells under lean electrolyte and limited K excess, reporting cycle life at practical areal loadings, energy density, Coulombic efficiency,^148^ impedance growth, gas generation,^146^ and safety metrics.^149,150^ Reports should consistently include parameters such as N/P ratios, electrolyte volume per areal capacity, stack pressure, and test temperature to enable cross-study comparison.^151,152^

### 3.5 Manufacturing and Sustainability

Transitioning from laboratory prototypes to industrially viable substrates requires scalable and cost-effective fabrication. Roll-to-roll coating, spray deposition, electrospinning, and chemical vapor deposition are promising techniques, but their compatibility with high-loading electrodes and uniform large-area coverage remains a bottleneck. Focus on processes compatible with roll-to-roll coating, dry-room operation, and cost-effective precursors. Incorporate cost models,^153^ supply chain assessments,^154^ solvent recovery,^155^ and life-cycle analysis,^156^ while developing strategies for recycling cathodes, K salts, separators, and current collectors.

### 3.6 Cross-Chemistry Transferability and Community Standards

Adapt insights from Li and Na systems while accounting for K-specific features such as ionic radius, solvation behavior, and SEI chemistry. Establish community-wide protocols, open data sets compliant with FAIR principles (Findable, Accessible, Interoperable, and Reusable),^157^ and unified reporting metrics.^158^ Publishing negative or marginal results should be encouraged to reduce redundant effort.

Accelerating the deployment of K metal anodes requires coordinated advances in mechanistic insights, multiscale modeling, realistic experimental designs, full-cell validation, sustainable manufacturing, and community-wide standards. Addressing the priorities outlined above will enable the transition from promising concepts to robust, cost-competitive, and scalable technologies.

While this review primarily focuses on K metal anodes, the underlying principles of substrate design show notable parallels with Li and Na counterparts. In all three systems, effective hosts mitigate dendritic growth by lowering local current density, improving metal–substrate binding, and directing uniform nucleation, while interfacial engineering strategies regulate SEI composition and mechanics. However, key distinctions arise from intrinsic material properties. K, with its larger ionic radius and weaker Lewis acidity, requires substrates with stronger potassiophilicity than those sufficient for Li or Na, and exhibits more pronounced volume fluctuations during deposition. Li metal benefits from relatively mature alloying and SEI-stabilization concepts, whereas Na and K highlight unique challenges in reversible alloying and SEI robustness under lean-electrolyte conditions. These differences underscore the need for tailored substrate chemistries rather than direct transference of Li-based designs.
